# The Effect of Liquid–Solid Interactions upon Nucleate Boiling on Rough Surfaces: Insights from Molecular Dynamics

**DOI:** 10.3390/ma16051984

**Published:** 2023-02-28

**Authors:** Chang Guo, Can Ji, Yalong Kong, Zhigang Liu, Lin Guo, Yawei Yang

**Affiliations:** 1 Energy Research Institute, Qilu University of Technology, Jinan 250014, China; 2 Electronic Materials Research Laboratory, Key Laboratory of the Ministry of Education, International Center for Dielectric Research, Shaanxi Engineering Research Center of Advanced Energy Materials and Devices, School of Electronic Science and Engineering, Xi’an Jiaotong University, Xi’an 710049, China

**Keywords:** engineered surface, nucleate boiling, molecular dynamics simulation

## Abstract

Despite the fact that engineered surface enabling remarkable phase change heat transfer have elicited increasing attention due to their ubiquitous applications in thermal management, the underlying mechanisms of intrinsic rough structures as well as the surface wettability on bubble dynamics remain to be explored. Therefore, a modified molecular dynamics simulation of nanoscale boiling was conducted in the present work to investigate bubble nucleation on rough nanostructured substrates with different liquid–solid interactions. Specifically, the initial stage of nucleate boiling was mainly investigated and the bubble dynamic behaviors were quantitively studied under different energy coefficients. Results shows that as the contact angle decreases, the nucleation rate increases, because liquid obtains more thermal energy there compared with that on less wetting surfaces. The rough profiles of the substrate can provide nanogrooves, which can enhance initial nucleate embryos, thereby improving thermal energy transfer efficiency. Moreover, atomic energies are calculated and adopted to explain how bubble nuclei are formed on various wetting substrates. The simulation results are expected to provide guidance towards surface design in state–of–the art thermal management systems, such as the surface wettability and the nanoscale surface patterns.

## 1. Introduction

Pool boiling, as a complex, non-equilibrium process with spatial inhomogeneity, depends on the nature of solid−liquid interactions which are not explainable using existing meso- and macro-scale approaches [1]. Nucleate boiling, as an efficient and controllable boiling stage, has found wide application in industry [2,3]. Nucleate boiling involves a slew of complex dynamic processes, including nucleation, development, coalescence, and detachment from a surface [4]. The critical heat flux (CHF) refers to the highest heat flux under certain surface superheat [5]. The substantial increases in heat flux and heat transfer efficiency accompany the rise of superheat. Maintaining nucleate boiling and enhancing CHF are important to the design of efficient and safe heat–dissipation systems [6]. Increases in the quantity of nucleation sites and the departing frequency and reduction of the departing radius are essential to improving the heat transfer [7,8].

At present, nanostructured surfaces (nanowires, for instance) have been produced to have adjustable and desirable specifications, which contributes to better control over surface morphologies [9]. Surface modifications such as variation of wettability and structures have internal correlations [10]. The nanostructures can make a hydrophilic coating surface more hydrophilic, and make a hydrophobic surface more hydrophobic. Generally, the small size of structures can affect bubble behaviors in the following ways [11,12,13]: (1) offering a larger number of nucleation sites; (2) facilitating expansion of bubbles by enlarging the contact areas; and (3) enhancing the departing frequency of bubbles favored by the capillary force [14]. Nanostructures can increase the area over which heat transfer occurs, which enables acquisition of more energy by liquid from solid surfaces and faster growth of bubble nuclei. Variation of wettability has been proven to be conflictive with improvement of the onset of nucleate boiling (ONB) and CHF at the same time, making it necessary to clarify the working mechanism underlying liquid–solid interactions on rough structure surfaces during nucleate boiling [15].

Boiling on solid surfaces containing nanoscale roughness has been studied in some experiments, including electropolished metal surfaces, silicon dioxide films, and vapor–deposited metal nanofilms [16,17,18]. However, the superheating reported by the experimental studies is much lower than the results calculated according to the classical nucleation theory. Among the reasons for this, a possible cause is that smooth solid substrates are not perfectly smooth in essence but contain some nanoscale and microscale structures [19]. Moreover, pre–existing gas may be present in cavities, thus enhancing formation of bubbles [20]. Surface morphologies play an important part in performance enhancement of boiling heat transfer, necessitating a deeper understanding of the mechanism underlying nucleation of nanostructures, and increasing thermal management efficiency in the engineering process. However, experiments fail to reveal all the detail of the early stage of bubble nucleation because physical mechanisms are complex and the accuracy of nanoscale observations is limited [21]. It remains to researchers to explore three-phase contact line motion and bubble expansion at the nanoscale [22].

Research at the molecular-scale appears to be promising for the modelling of pool boiling [23]. This is because the out-of-equilibrium nature of boiling and the molecular interaction effects are acquired at the atomistic scale, and there is no need to set closure assumptions [24]. Molecular dynamic simulations are an approach that has found extensive application to interfacial research because of the high resolution and low cost [25]. Molecular modelling of pool boiling provides double advantages: firstly, it offers insights and helpful information suitable for models at a larger scale; secondly, revealing the atomistic nature of phase change allows one to explore pivotal phenomena and basic principles. It is worth noting that many studies have been conducted on explosive boiling and nucleate boiling induced by heating solid walls underneath the liquid [26,27]. Smakulski et al. have summarized and made comparison of three techniques for increasing heat flux by taking construction, reliability, and feasibility into consideration [28,29]. Influences of surface wettability on explosive boiling have also been explored in liquids in the fast heating process. According to previous research, strong–wettable surfaces are conducive to explosive boiling. Molecular dynamics (MD) simulations of explosive boiling have also been conducted on the thin layer of liquid argon on nanostructured solid surfaces of various wettabilities [30,31,32]. The research revealed that strong wettable surfaces enable a far higher rate of energy transfer compared with those in other cases. The present literature review indicates that the three common methods to induce bubble nucleation mainly include direct control of liquid temperature, creation of an under pressure, and liquid superheating based on solid surfaces.

Although numerous studies have been reported, the role of solid–liquid interactions in nanobubble nucleation still calls for systematic exploration. First, the boiling surface is not strictly smooth. The randomly distributed rough nano–structures [33,34] instead of artificial rule structures [35] should be discussed to provide better guidance of surface designation, especially on such a small simulation scale. Secondly, bubbles are expected to exhibit various dynamic behaviors during different environment pressures [36,37]. However, the fixed volume method has usually been applied in literatures, which means that the change of the fluid temperature induced by the heat transfer results in a change of the system pressure [38]. The system pressure during the phase–change process should be taken into consideration. Therefore, the present research contributes to highlight effects of rough nanostructures, surface wettability, and system pressure in driving and controlling formation of nanobubbles by conducting systematic parametric analysis.

A nucleate boiling model of liquids on a rough structural surface is constructed in the present work. The research simulates phase changes in the Lennard Jones (LJ) system adopting non-equilibrium molecular dynamics. The LJ system is investigated mainly because of the low computational cost, which allows investigation under numerous operating conditions, without loss of generality. The interface evolution is directly observed. The mechanism of the liquid–solid interactions on the heat transfer is explained from the microscopic perspective. The remainder of the paper is organized as follows: detailed numerical information and operation conditions of the system studied are introduced; then, the main results are provided and discussed; finally, the last section provides conclusions and suggestions for future research.

## 2. Simulation Method

### 2.1. Simulation Model

**Figure 1:**
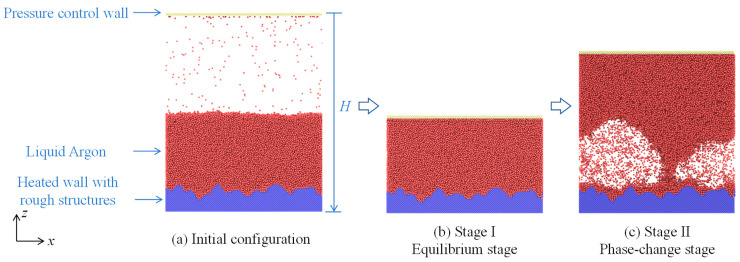
Simulation system set-up and simulation procedures. (**a**) Initial configuration of the simulation system; (**b**) Stage I of the simulation: Equilibrium stage; (**c**) Stage II of the simulation: Phase–change stage.

Figure 1a displays the schematic map of the simulated system, which initially consists of the following three parts: argon liquid, argon vapor, and a nanostructured copper surface. The argon is chosen as the simulation medium since it is the conventional working fluid in most MD simulations. Copper is selected for being one of the most attractive materials for heat-transfer studies due to its high thermal conductivity. The initial simulation box measures 300 Å (*x*) × 50 Å (*y*) × 432 Å (*z*). The pseudo-two-dimensional method that is advantageous in capturing main physical processes of boiling at low computational expense is used, though it maintains a wide system along the *x*- and *z*-directions. Solid surfaces are set at the top and bottom of the simulation box and they are generated in face–centered cubic lattices (lattice constant: 0.35 nm). The bottom wall is set as the heat source during the boiling process. Rough structures are generated on the solid surface, the details of which are elaborated upon in Section 2.2. The upper wall is set to control the system pressure, as explained in Section 2.3. Argon atoms are placed above the rough substrate and are produced in simple cubic lattices (lattice constant: 0.52 nm). The periodic boundary conditions are applied to *x*- and *y*-directions, while the fixed boundary condition is applied to the *z*-direction. The atomic interactions in the present study are characterized by the pairwise LJ potential [12]

(1) $$\phi \left(r_{ij}\right)=4\varepsilon_{ij}\left[{\left(\frac{\sigma_{ij}}{r_{ij}}\right)}^{12}-{\left(\frac{\sigma_{ij}}{r_{ij}}\right)}^{6}\right],r_{ij}\leq r_{c}$$

where *σ_ij_* and *ε_ij_* separately denote distance and energy parameters of two generic atoms *i* and *j* with a distance *r_ij_*, and *r*_c_ represents the cut–off distance. Interaction potential between argon atoms (Ar) and copper atoms (Cu) has correlations with wettability at solid–liquid interfaces, which can be calculated thus:

(2) $$\varepsilon_{\mathrm{Cu}-\mathrm{Ar}}=\alpha \sqrt{\varepsilon_{\mathrm{Cu}}\varepsilon_{\mathrm{Ar}}},$$

(3) $$\sigma_{\mathrm{Cu}-\mathrm{Ar}}=\frac{\sigma_{\mathrm{Cu}}+\sigma_{\mathrm{Ar}}}{2},$$

where the energy coefficient *α* is introduced to adjust the substrate wettability. The greater the *ε*_Cu–Ar_ is, the higher the energetical intensity of atomic Ar–Cu interactions. Potential energy parameters are set in Table 1.

### 2.2. Rough Surface Construction

According to the classical heterogeneous nucleation theory, grooves on substrates are sites desirable for breeding bubble nuclei. Solid surfaces are not perfectly smooth but contain defects, so rough substrates are considered in the present study. To construct rough surfaces with random distributions, the following generalized W–M (Weierstrass–Mandelbrot) function involving several variables is applied [39]:

(4) $$z\left(x,y\right)=C\sum_{m=1}^{M}\sum_{n=0}^{n_{\max}}\gamma^{\left(Ds-3\right)n}\cdot \left\{\cos\Phi_{m,n}-\cos\left[\frac{2\pi \gamma^{n}\sqrt{x^{2}+y^{2}}}{l_{\max}}\cdot \cos\left(\tan^{-1}\left(\frac{y}{x}\right)-\frac{\pi m}{M}\right)+\Phi_{m,n}\right]\right\}$$

It is superimposed on two-dimensional fractal profiles, namely, *M* ridges, which form surface morphologies with random phases. The random phase array Φ*_m,n_* is several stochastic values with random distributions within [0, 2π], which prevents cosine functions coinciding with the geometrically increased frequency. *γ* (*γ* > 1) as a parameter controlling frequency density, is set to 1.5 in the present study. *l*_max_ represents sample size that is measured in the length unit; *D*_s_ denotes the fractal dimension (2 < *D*_s_ < 3) of surfaces. Contributions of high and low–frequency components to surface profiles are determined by the value of *D*_s_, which is set to 2.8. *C* indicates a scaling factor that determines the mean amplitude of cosine waves, as given by [17]

(5) $$C=l_{\max}{\left(\frac{G}{l_{\max}}\right)}^{D_{s}-2}\frac{In\gamma^{1/2}}{M},$$

where *G* represents the roughness constant because of correspondence of greater *G* to rougher surfaces. The research applies *G* = 0.5 to produce rough substrates. Then, based on a smooth surface, the number of copper atoms is increased or decreased in accordance with relative locations of various copper lattice sites with values of the W–M function. A custom MATLAB script is firstly written to generate coordinates of atoms that compose the rough substate controlled by the W–M function. Then the coordinates of the atoms are read by the LAMMPS code. The resulting solid substrate is shown in Figure 1.

### 2.3. Pressure Control Method

**Figure 2:**
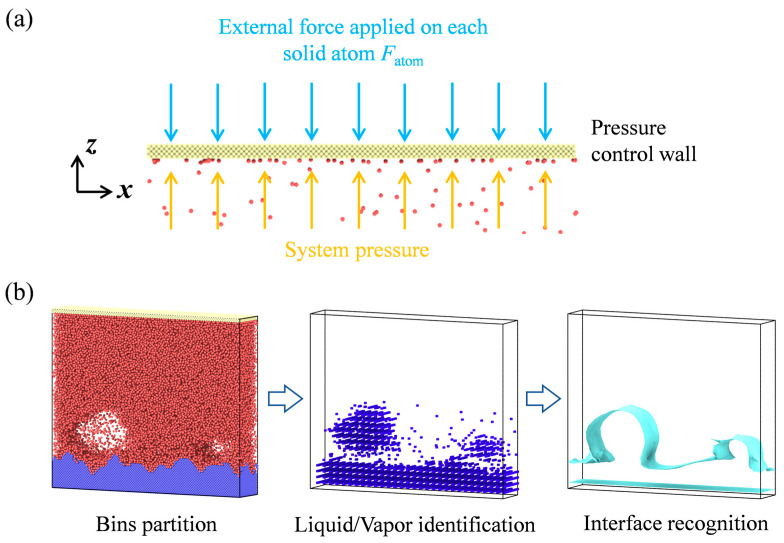
(**a**) Pressure control method; (**b**) Bubble identification.

Usually, in the MD simulation of pool boiling, the size of the simulation box remains unchanged, however, during the boiling process, the liquid will undergo a gas–liquid phase change under the heating of the wall, and the system pressure will change if the simulation area remains unchanged, which will influence the whole boiling process. Therefore, a systemic pressure-control method is proposed herein to keep the pressure in the simulated area constant in the boiling process. Specifically, a control wall is set above the simulation area, as shown in Figure 2a. In the simulation process, an external force is applied to each solid particle on the control wall. The applied external force in the *z*-direction is balanced with the pressure applied by the system to the pressure control wall in the *z*-direction, to keep the whole system pressure constant and avoid the influence of pressure change on the bubble dynamics. The force applied on every single atom of the pressure–control wall *F*_atom_ is calculated by

(6) $$F_{\mathrm{atom}}=\frac{F_{\mathrm{total}}}{N_{\mathrm{up}}}=\frac{p\times S_{xy}}{N_{\mathrm{up}}},$$

where *F*_total_ is the total force applied on the pressure–control wall, *N*_up_ is the number of atoms on the pressure–control wall, *p* denotes the pressure set in the simulation, which is 1 bar in the present work, and *S_xy_* denotes the cross–section area on the *x–y* plane. It should be noted here that the force applied on the pressure–control wall is only used to control the system pressure during the phase-change process. The size of the simulation box in the z-direction is not fixed, but can change along with the system pressure. Due to this, the force applied will only affect the pressure control wall, having no effect on argon atoms.

### 2.4. Surface Wettability Evaluation

The solid–liquid interaction strength, namely the interfacial wettability, can be regulated through varying the energy parameter between the argon atom and the copper atom. Therefore, an energy coefficient *α* is introduced to represent the solid–liquid interaction. In order to characterize the solid–liquid interaction, the relationship of surface wettability, i.e., the contact angle of argon atoms on the copper surface, with the energy coefficient should be explored first. Specifically, a spherical argon droplet, the diameter of which is 6 nm, is first put on the bottom solid wall of the simulation box having dimensions of 16 nm × 5 nm × 20 nm. Separate contact angles are then simulated in the constant–energy, constant–volume ensemble (NVE), also known as the microcanonical ensemble, during which all atoms are kept at 80 K by using a Langevin thermostat. The simulation lasts for 1 ns to ensure that the droplet has been sufficiently equilibrated. For equilibrium droplets on the copper surface, the droplet boundary is defined generally through algebraic circle fitting and the contact angle can be measured graphically. The energy coefficient of solid and fluid atoms, *α*, determines solid–liquid interactions and it is set explicitly and appropriately to enable different surface wettabilities. The contact angles are measured in 12 cases of various surface wettabilities (*α* = 0.02, 0.04, 0.06, 0.2, 0.4, 0.8, 1.5, 2, 2.5, 3, 4, 5). The contact angle (CA) calculation procedure is as follows [40]: (a) The computational box is discretized into bins of square cross sections in the *x*−*z* plane, and the entire length of the computational box in the *y* direction is the depth of the bins. The resolution of the bins was set as 0.8 × 0.8 Å^2^. The position of each atom of the tested group is stored in the appropriate bin for every snapshot, and the mass density is calculated as the average over time of the bins count per unit volume. (b) The averaged mass density of bins is plotted as contour maps, and the droplet interface is defined by the contour line in which *ρ*(*x*, *y*) = *ρ*_l_/2, where *ρ*_l_ is the fluid density. (c) The coordinates of the surface position and the droplet interface are obtained and fitted using the equation of a circle. (d) Finally, the fitted equation is plotted, and the contact angle is measured.

### 2.5. Simulation Procedures

MD simulations are performed using the large–scale atomic/molecular massively parallel simulator (LAMMPS). The simulation process is divided into two stages: the equilibrium stage (Stage I, Figure 1b), and the phase-change stage (Stage II, Figure 1c). A Nose−Hoover thermostat with a characteristic relaxation time constant of 100Δ*t* to achieve equilibrium of the initial configuration of the system in an NVT ensemble (constant molecular number (N), constant volume (V), and constant temperature (T)) is used for updating positions and velocities of atoms in each time step. Therein, Δ*t* represents the value of time steps, which is set to 0.005 ps by referring to Refs. [17,25]. The equilibration lasts for 5000 ps to bring the system to the target temperature *T*_cold_ = 80 K. After the equilibration stage, the temperature of bottom surface rises to *T*_heat_ = 160 K. The temperature is set for the purpose of collecting the evaporate argon atoms and saving compactional source. Heat is provided from the bottom by retaining the solid substrate thermostatic control afforded by the Langevin thermostat. The standard velocity Verlet integrator algorithm is adopted for time–integrating the equation of motion of atoms. Simulations last for 5000 ps in the microcanonical ensemble. It must be noted that a harmonic spring force with a spring constant of 100 is used to tether solid atoms at their equilibrium positions in the entire simulation process. This prevents penetration and translational movements of liquid–phase atoms in the simulation.

### 2.6. Data Post Processing

Graphical images of the molecular systems are generated using an Open Visualization Tool (OVITO). For the sake of investigating heat transfer during nanoscale phase change, heat flux is calculated thus [41]:

(7) $$q=\frac{1}{V}\left[\sum_{i}e_{i}u_{i,z}+\sum_{i<j}\left(f_{ij}\cdot \left(u_{i}+u_{j}\right)\right)x_{ij}\right],$$

where *e_i_* denotes energy per atom (kinetic and potential included); *u_i,z_* represents the velocity of an atom along the *z-*direction; *f_ij_* is the pair-wise force vector of atoms *i* and *j*; *u_i_* and *u_j_* separately denote velocity vectors; *x_ij_* is a position vector. A simple criterion based on the instantaneous positions of particles (Figure 2b) is used for quantitative description of bubbles. The simulation domain is divided into three-dimensional cells of 0.2 nm, which are deemed to be “voids” if there are no argon atoms in this domain. Small voids continue to grow and coalesce to form large bubbles defined by the sum of connected voids. Thus, the vapor–liquid interface is formed and can be recognized and calculated by OVITO.

## 3. Results and Discussion

### 3.1. Effect of Argon–Copper Interactions on the Contact Angle

The relationship between the contact angle and energy coefficient is simulated (Table 2): the trend in the data is to a gradual decrease in the contact angle as the energy coefficient increases, which leads to the nonwetting-to-superwetting transition of surface wettability. After the energy coefficient reaches 1.5, the surface wettability remains unchanged as the surface is superwetting.

### 3.2. Preliminary Visualization of Bubble Dynamics

**Figure 3:**
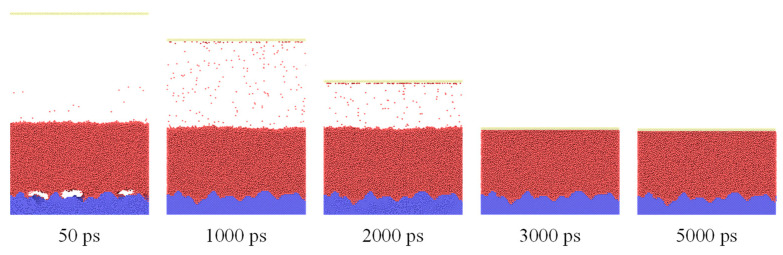
Snapshots of the system at the equilibrium stage.

Based on the aforementioned simulation system and approaches, simulations are performed in 12 cases. The simulation is conducted in two stages. Figure 3 shows the snapshots of the system at the equilibrium stage, which shows that the simulation box has a decreased height over time. This is because the pressure control method is applied. There are two types of forces applied to the atoms of the upper wall: one comes from the system pressure, which is in the *z*-direction; the other is the external force *F*_atom_ in the –*z*-direction. Therefore, the upper wall moves due to the unbalanced forces, reaching a stable state in the equilibrium position.

**Figure 4:**
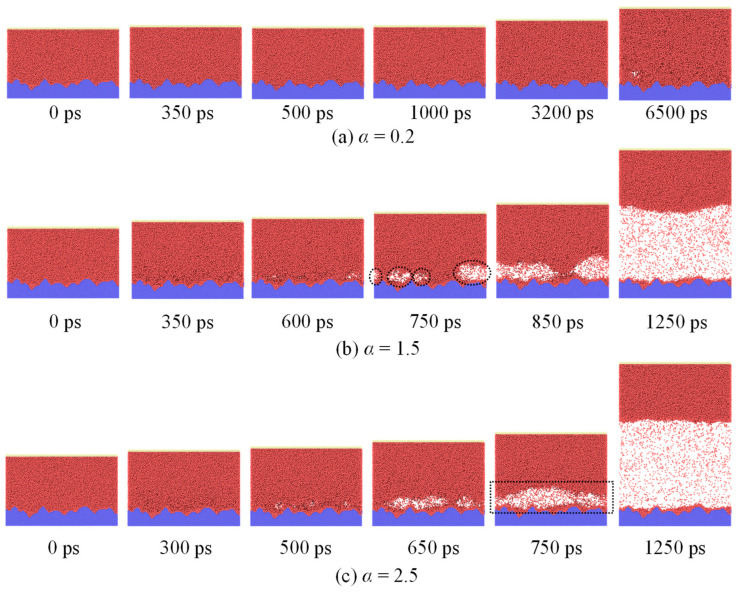
Snapshots of three typical phase–change processes of liquid argon on nanostructured surfaces with different solid–liquid interactions. (**a**) *α* = 0.2; (**b**) *α* = 1.5; (**c**) *α* = 2.5.

After the system reaches equilibrium, the bottom wall is heated, and the phase–change process starts. Different phenomena are found under variation of the surface wettability. Figure 4 illustrates snapshots of three representational cases (Cases 4, 7, and 9). The upper wall moves slowly as the argon is heated by the bottom wall (Figure 4a), however, there is no boiling observed when *α* is 0.2.

**Figure 5:**
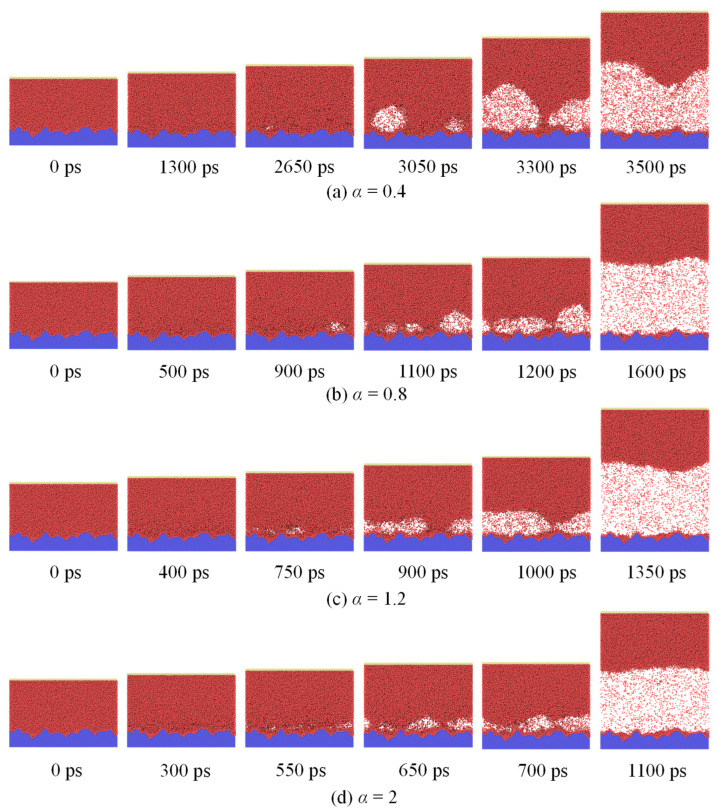
Snapshots of nucleate boiling of liquid argon on nanostructured surfaces with different solid–liquid interactions. (**a**) *α* = 0.4; (**b**) *α* = 0.8; (**c**) *α* = 1.2; (**d**) *α* = 2.

In contrast, nucleate boiling phenomena is observed when *α* is 1.5. Moreover, representative snapshots of nucleate boiling of liquid argon on nanostructured surfaces with different solid–liquid interactions are shown in Figure 5. At first, despite the random appearance and disappearance of voids in space and time, these voids always fail to form bubbles because their size are smaller than the critical size of nuclei throughout the simulation process. It should be noted here that if a void can continue to grow into a bubble, then the initial observed size is defined as the critical of nuclei in the present study. As time continues, bubble embryos generate in an activated site, i.e., nucleation site, which are marked with dashed circles in Figure 4b. Bubble embryos undergo rapid expansion because of heat transfer of liquid surrounding the bubble, forming a dry area below the bubble surrounded by the three-phase contact line. At about 850 ps, it is found that many little voids coalesce to form an apparent bubble. If buoyancy exceeds surface tension, the liquid is likely to rewet dry sites and a bubble gradually departs from the surface. Attention should be paid to the crystallization of liquid atoms close to the surface heated, which surround two liquid-atom layers that need to be regarded as solid. The three-phase contact line does not have direct contact with substrates. Then, it continues to develop as a thermodynamically stable bubble until becoming a vapor film. Hence, the growth process of a bubble can be portioned into two stages: slow void growth and coalescence, and rapid growth of the bubble.

In Case 9 (*α* = 2.5), voids form above the surface and develop rapidly to a vapor film (750 ps), followed by complete isolation of the film from the surface, that is, occurrence of explosive boiling.

**Figure 6:**
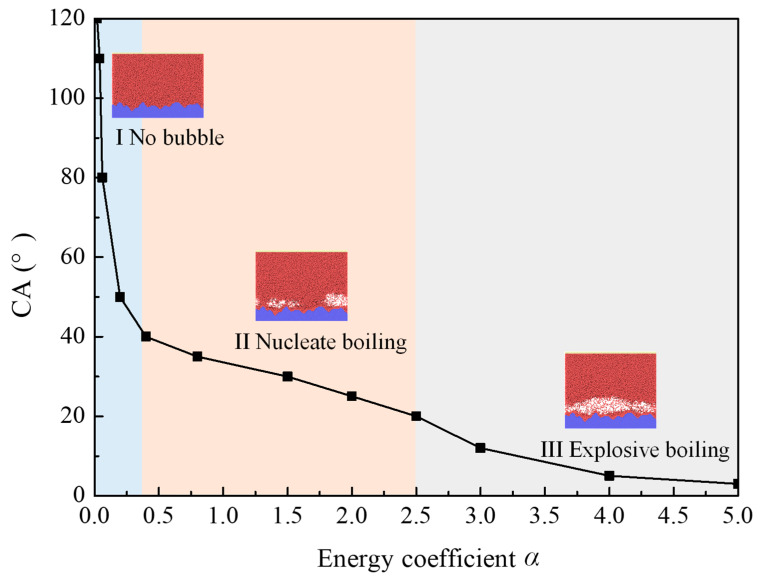
Phage–change diagram for the different energy coefficient. The wall superheating equals 80 K.

These results suggest presence of a reasonable wettability range allowing occurrence of stable bubbles. According to results in all 12 cases, the wettability in Figure 6 is roughly differentiated by proposing three regions: Regions I (*α* < 0.4), II (0.4 ≤ *α* < 2.5), and III (*α* ≤ 2.5), which separately represent cases of no bubble, all cases of nucleation boiling, and all cases of explosive boiling. Hence, it is a delicate matter to choose the surface wettability. There is a reasonable wettability range (Region II) for the appearance of stable bubbles. It is worth noting here that values in this range are only suitable for the situation discussed in the present work. However, the changing trend of the boiling mode with the solid–liquid interactions applied to global situations.

### 3.3. Quantitative Research into the Effect of Solid–Liquid Interactions

According to the above analysis, it is proven that the formation probability of voids above weak wettable surfaces is higher compared with those above strong wettable surfaces before nucleation. The continuous heat transfer from surface to liquid enables formation of stable bubbles by these voids, which occurs only when surface wettability is in the range of 0.4 ≤ *α* ≤ 2.5 (Section 3.2). In this section, the bubble growth, and the heat–transfer performance on surfaces of different wettability are quantitatively investigated based on methods introduced in Section 2.

**Figure 7:**
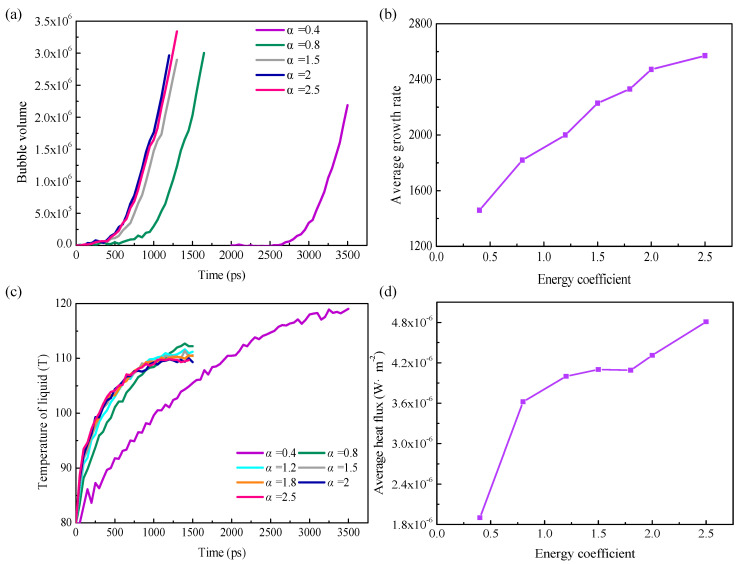
Quantitative investigations of the boiling process with different solid–liquid interactions. (**a**) The time evolution of the bubble growth curve; (**b**) The average bubble growth rate with different solid–liquid interactions; (**c**) The time evolution of temperature in the liquid film near the bottom wall; (**d**) The average heat flux with different solid–liquid interactions.

Figure 7a shows the temporal evolution of the bubble volume. It can be obtained that the entire boiling processes is classified into two stages based on the volume change trend: a slow nucleation stage and a rapid growth stage to form stable bubbles. Naturally, this shares some similarity with the homogeneous nucleation process in previous research, and the slow nucleation stage turns to the rapid growth stage only when critical nuclei are formed. The waiting time *t*_w_ is defined as the simulation duration from the beginning to the moment for appearance of critical nuclei, and the inception time *t*_in_ is the time taken for formation of the vapor film from the non–equilibrium stage. Figure 7a indicates that the time taken by voids to grow to the size of critical nuclei shortens as the surface wettability increases in the formation process of critical nuclei.

At the nanoscale, greater wettability corresponds to lower interfacial thermal resistance and therefore more easy nucleation. A bubble continues to steadily grow when its volume reaches the critical level, that is, the bubble enters the rapid growth stage. Considering this, studying the rate of bubble growth in this stage is important. It is worth noting that here the average growth rate *J*_g_ indicates a single bubble, which can be expressed as

(8) $$J_{g}=\frac{\Delta V}{t_{in}-t_{w}},$$

where Δ*V* represents the difference of the volume of the greatest bubbles before departing from the substrate with the critical volume; *t*_in_ − *t*_w_ is the time taken by a bubble to grow from critical nuclei to a vapor film. Figure 7b depicts the increasing growth rate of bubbles as the surface wettability rises.

Figure 7c,d illustrates time evolution of the liquid temperature and heat fluxes under different solid–liquid interactions on rough surfaces. The liquid rises as the boiling process continues, and the heat flux increases with the increasing energy coefficient. To explore the reasons behind this, density and temperature contours are also plotted. Classical nucleation theory reveals that local regions of low density will be formed in the liquid due to density fluctuations attributed to disordered molecular movement. These regions of low density are regarded as voids, where nucleation is initiated, and so it is of significance to explore how surface wettability affects formation of voids. It should be noted here that the temperature of liquid with *α* equals 0.4 has an apparent fluctuation compared with the other cases. The reason might be the relatively low bubble growth rate when *α* equals 0.4. As shown in Figure 5a, it takes around 3500 ps for the bubble growing large enough to depart. Bubbles encounter fluctuation with each other during this period, resulting in the fluctuated temperature curve.

**Figure 8:**
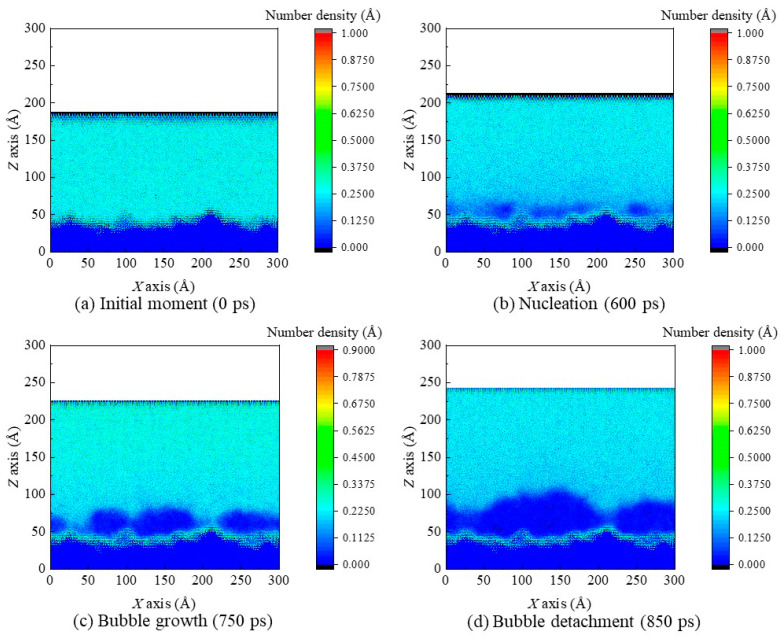
The number density contours of the case *α* = 1.5 at (**a**) the initial moment, (**b**) the nucleation moment, (**c**) a certain bubble growth moment, and (**d**) the bubble detachment moment.

The simulation domains are discretized into meshes measuring 2 Å (*x*) × 2 Å (*z*). Number density contours of the case *α* = 1.5 at the initial moment, the nucleation moment, a certain bubble growth moment, and the bubble detachment moment are plotted (Figure 8). The density in navy blue regions is the lowest (nearly zero), so there is the highest probability for formation of voids. In time, the number of the navy–blue regions increases; it is worth noting here that, there are many more voids on this rough nanostructured surface compared with that on smooth surfaces. This is because the rough surface provides more nanogrooves to generate void. Thus, the voids grow and merge with each other (i.e., nucleation), which also coincides with the snapshots in Figure 5.

**Figure 9:**
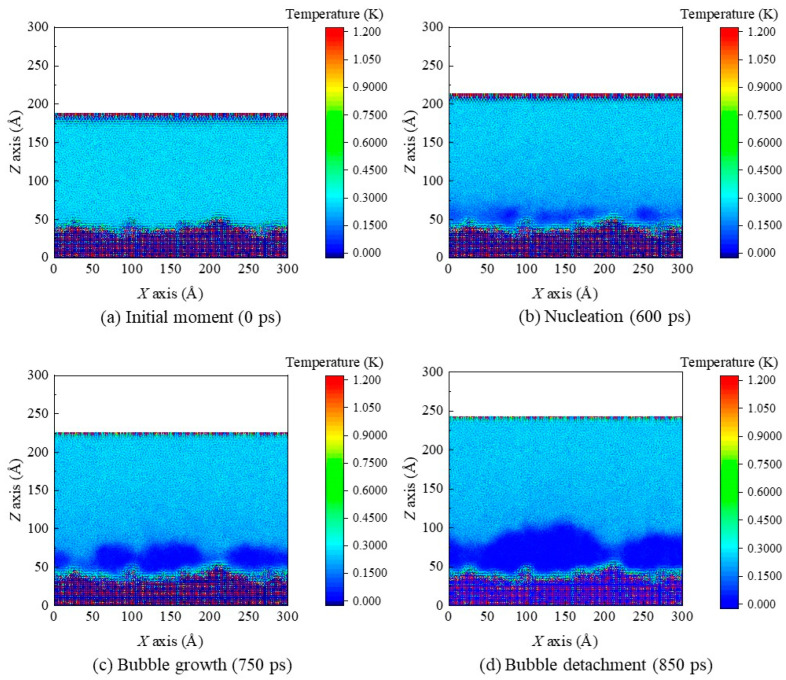
The temperature contours of the case *α* = 1.5 at (**a**) the initial moment, (**b**) the nucleation moment, (**c**) a certain bubble growth moment, and (**d**) the bubble detachment moment.

Figure 9 displays contours for argon temperature under *α* = 1.5 on the *x–z* plane in representational time steps. Uniform temperature contours with an average value of 80 K are observed in the initial time step. Then, the temperature rises gradually in liquid regions during heat transfer from solid surfaces to liquid regions. At the initial moment, no stable bubble appears, with uniform temperature distribution in liquid regions. As stable bubbles are gradually formed and develop, non–uniform temperature distribution begins to appear in liquid regions. It can be seen, from Figure 9b, that the corresponding regional temperature of voids decreases gradually.

According to the above results, the quantity of voids in liquid above the surfaces gradually increases, and the probability of nucleation also increases with the enhanced solid–liquid interaction. Strongly wettable surfaces have higher heat transfer efficiency compared with weakly wettable ones, and stable bubbles occur above strongly wettable surfaces before those above weakly wettable ones. As a result, more energy is acquired by argon on strongly wettable surfaces compared with that on weakly wettable ones. Because the nucleation process of bubbles occurs at the nanoscale, the surface effect is non-negligible and it even functions as the dominant factor for the nucleation process.

### 3.4. Mechanisms behind Pool Boiling with Different Solid–Liquid Interactions

**Figure 10:**
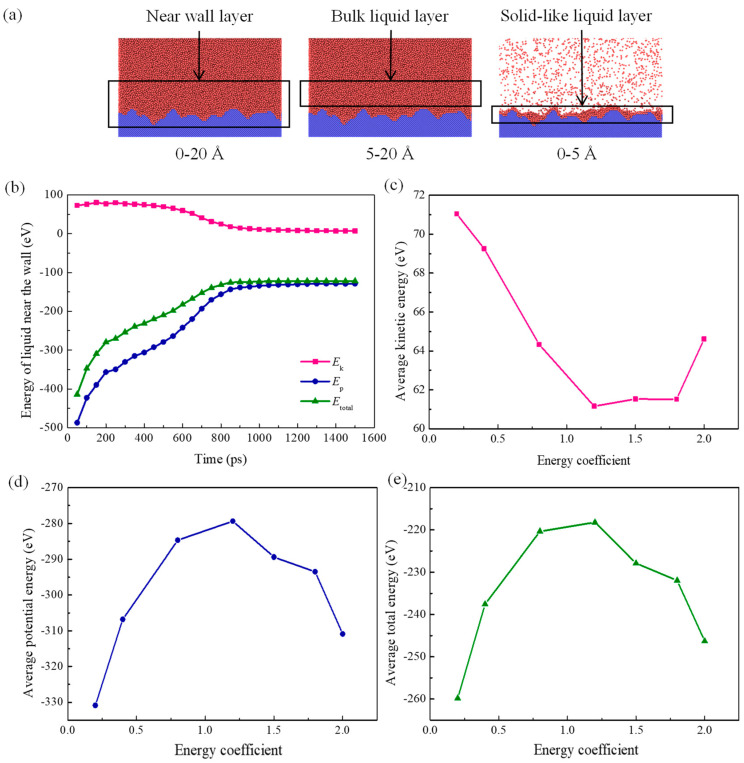
(**a**) The schematic of the liquid configuration in the near-wall region (from *z* = 0 Å to *z* = 20 Å), the bulk liquid layer (from *z* = 5 Å to *z* = 20 Å), and the solid-like liquid layer (from *z* = 0 Å to *z* = 5 Å); (**b**) The time evolution of different energies in the near-wall layer when *α* = 1.5; The average kinetic energy (**c**), average potential energy (**d**), and average total energy (**e**) with different solid–liquid interactions.

**Figure 11:**
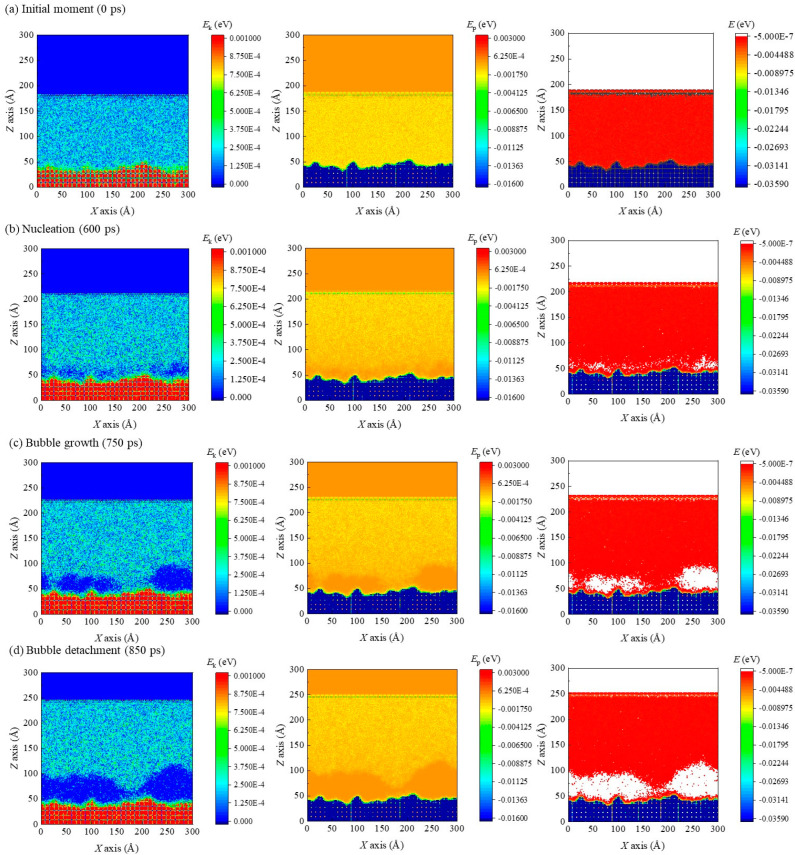
The kinetic energy contours (left side), the potential energy contours (middle), and the total energy contours (right side) of the case *α* = 1.5 at (**a**) the initial moment, (**b**) the nucleation moment, (**c**) a certain bubble growth moment, and (**d**) the bubble detachment moment.

It is worth noting that intense interatomic solid–liquid interactions on super–wetting surfaces are likely to inhibit atomic evaporation in solid-like liquid layers. This is always limited to substrate surfaces (right–hand panel, Figure 10a). This means that solid–like liquid atoms do not in fact participate in nucleation of bubbles. Therefore, it is necessary to divide that near–wall liquid film (from *z* = 20 Å to *z* = 40 Å in the current study) into two individual regions when analyzing energy barriers of nucleation: one is the solid-like liquid layer (from *z* = 20 Å to *z* = 25 Å), and the other is the near-wall bulk liquid layer (from *z* = 25 Å to *z* = 40 Å) (Figure 11a). The former is projected to have thickness of 5 Å according to snapshots and contours obtained above. Furthermore, the temporal evolution of kinetic energy, potential energy, and total energy in the near-wall layer in the case that *α* is 1.5 and the average energy under various solid–liquid interactions in the near-wall layer are calculated separately and presented in Figure 10b–e. Contours for kinetic energy, potential energy, and total energy in the case *α* = 1.5 at the initial moment (0 ps), nucleation (600 ps), moment of certain bubble growth (750 ps), and bubble detachment moment (850 ps) are also plotted (Figure 11).

The potential energy in the near-wall layer is found to dramatically rise with the energy coefficient. The mean potential energy barrier for liquid films in the near-wall region always increases with enhanced solid–liquid interactions, which is mainly attributed to the solid-like liquid layer. This is also confirmed by comparison of contours for potential energy. According to the above analysis, the solid-like liquid layer has a much higher number density compared with bulk liquid film, leading to a shortened distance of liquid atoms in this layer and therefore greater average potential energy. The effect of solid–liquid interactions upon heat transfer during nucleate boiling on superwetting surfaces mainly arises from changes in heat transfer efficiency at solid–liquid interfaces. It is fascinating that the near-wall bulk liquid layer plays the dominant part in the analysis of potential energy barriers, to which close attention needs to be paid.

With the temperature rise of bottom walls from equilibrium to target values, most of the absorbed thermal energy is transformed into atomic kinetic energy in the solid-like liquid layer, leading to a significant increment in the overall temperature. Energy is found to undergo only small changes in the solid-like liquid layer, while most thermal energy transferred from the heated wall is absorbed by the near-wall bulk liquid layer. Moreover, it is worth noting that thermal energy absorbed by the bulk liquid layer is mainly used for overcoming potential energy barriers of liquid atoms regardless of the heating stage. The result also verifies that this near-wall bulk liquid layer is significant for analyzing energy barriers to nucleation.

## 4. Conclusions

The bubble dynamics of nucleate boiling on the rough nanostructured surface with different wettability was analyzed by conducting a modified molecular dynamics method. The following key findings are drawn from the simulated results. Bubbles can be observed if the energy coefficient *α* between surfaces and fluid atoms is in a reasonable range (0.4 ≤ *α* ≤ 2.5). Explosive boiling would be formed if the surface were superwetting. Void generation and bubble growth rate were increased by increased liquid–solid interactions. The rough structured surfaces were reported to generate new contact lines on structures, which included more microlayers and the heat transfer was enhanced as a result. The simulation results are expected to reveal boiling phenomena in nanoscale and provide guidance towards surface design in state-of-the-art thermal management systems.

## Figures and Tables

**Table 1:** Potential energy parameters.

Inter–Particle Interaction	Potential Energy Parameter	
	*ε*/eV	*σ*/Å
Ar–Ar	0.0104	3.405
Ar–Cu_bottom_	Variable	2.87
Ar–Cu_up_	0.02	2.87
Cu_bottom_–Cu_bottom_, Cu_up_–Cu_up_	0.406	2.338
Cu_bottom_–Cu_up_, Cu_bottom_–Cu_up_	0	0

**Table 2:** Surface wettability under different solid–liquid interactions.

	Energy Coefficient *α*	Energy Parameter *ε*_Ar–Cu_ (eV)	Contact Angle CA (°)	Surface Wettability
Case 1	0.02	0.0013	120	Nonwetting
Case 2	0.04	0.0026	110	Nonwetting
Case 3	0.06	0.0039	80	Wetting
Case 4	0.2	0.013	50	Wetting
Case 5	0.4	0.026	40	Wetting
Case 6	0.8	0.052	35	Wetting
Case 7	1.5	0.0975	30	Wetting
Case 8	2	0.13	25	Superwetting
Case 9	2.5	0.1625	20	Superwetting
Case 10	3	0.195	12	Superwetting
Case 11	4	0.26	5	Superwetting
Case 12	5	0.325	3	Superwetting

## Data Availability

Not applicable.

## Nomenclature

CHF	The critical heat flux
ONB	The onset of nucleate boiling
MD	Molecular dynamics
LJ	Lennard Jones
Ar	Argon
Cu	Copper
W–M	Weierstrass–Mandelbrot
NVE	the constant–energy, constant–volume ensemble
LAMMPS	The large-scale atomic/molecular massively parallel simulator
NVT	constant molecular number, constant volume, and constant temperature ensemble
OVITO	Open Visualization Tool
CA	Contact angle
*φ*	The Lennard–Jones potential
*σ*	The distance parameter
*ε*	The energy parameter
*r_ij_*	The distance between two atoms *i* and *j*
*r*_c_	The cut–off distance
*Φ*_m,n_	Stochastic values with random distributions within [0, 2π]
*γ*	The parameter controlling frequency density
*l*_max_	The sample size measured in the length unit
*D*_s_	The fractal dimension of surfaces
*G*	The roughness coefficient for scaling the height of functions
*F*_atom_	The force applied on every single atom of the pressure control wall
*F*_total_	The total force applied on the pressure control wall
*N*_up_	The number of atoms on the pressure control wall
*p*	The pressure set in the simulation
*S_xy_*	The cross–section area on the *x–y* plane
*α*	The energy coefficient of the solid–liquid interaction
Δ*t*	The timesteps
*T*_cold_	The initial temperature of the simulation
*T*_heat_	The target temperature of the bottom surface
*q*	The heat flux
*e_i_*	The energy per atom (kinetic and potential included)
*u_i,z_*	The velocity of an atom along the *z*-direction
*f_ij_*	The pair–wise force vector of atoms *i* and *j*
*u*	A velocity vector
*x_ij_*	A position vector
*t*_w_	The time from the beginning to the moment of the critical nuclei appearance
*t*_in_	The time taken for formation of the vapor film from the non-equilibrium stage
*J*_g_	The average growth rate of a single bubble
Δ*V*	The volume difference of the greatest bubbles before departing from the substrate
